# Iowa Mutant Apolipoprotein A-I (ApoA-I_Iowa_) Fibrils Target Lysosomes

**DOI:** 10.1038/srep30391

**Published:** 2016-07-28

**Authors:** Hirokazu Kameyama, Hiroyuki Nakajima, Kazuchika Nishitsuji, Shiho Mikawa, Kenji Uchimura, Norihiro Kobayashi, Keiichiro Okuhira, Hiroyuki Saito, Naomi Sakashita

**Affiliations:** 1 Department of Molecular Physical Pharmaceutics, Institute of Biomedical Sciences, Tokushima University Graduate School, 1-78-1 Shomachi, Tokushima 770-8505, Japan; 2 Department of Molecular Pathology, Institute of Biomedical Sciences, Tokushima University Graduate School, 3-18-15 Kuramoto-cho, Tokushima 770-8503, Japan; 3 Department of Biophysical Chemistry, Kyoto Pharmaceutical University, 5 Nakauchi-cho, Misasagi, Yamashina-ku, Kyoto 607-8414, Japan; 4 Department of Biochemistry, Nagoya University Graduate School of Medicine, 65 Tsurumai-cho, Showa-ku, Nagoya 466-8550, Japan; 5 Department of Bioanalytical Chemistry, Kobe Pharmaceutical University, 4-19-1 Motoyama-Kitamachi, Higashinada-ku, Kobe 658-8558, Japan

## Abstract

The single amino acid mutation G26R in human apolipoprotein A-I (apoA-I_Iowa_) is the first mutation that was associated with familial AApoA1 amyloidosis. The N-terminal fragments (amino acid residues 1–83) of apoA-I containing this mutation deposit as amyloid fibrils in patients’ tissues and organs, but the mechanisms of cellular degradation and cytotoxicity have not yet been clarified. In this study, we demonstrated degradation of apoA-I_Iowa_ fibrils via the autophagy-lysosomal pathway in human embryonic kidney 293 cells. ApoA-I_Iowa_ fibrils induced an increase in lysosomal pH and the cytosolic release of the toxic lysosomal protease cathepsin B. The mitochondrial dysfunction caused by apoA-I_Iowa_ fibrils depended on cathepsin B and was ameliorated by increasing the degradation of apoA-I_Iowa_ fibrils. Thus, although apoA-I_Iowa_ fibril transport to lysosomes and fibril degradation in lysosomes may have occurred, the presence of an excess number of apoA-I_Iowa_ fibrils, more than the lysosomes could degrade, may be detrimental to cells. Our results thus provide evidence that the target of apoA-I_Iowa_ fibrils is lysosomes, and we thereby gained a novel insight into the mechanism of AApoA1 amyloidosis.

## 

Apolipoprotein A-I (apoA-I), the major protein in high-density lipoproteins, plays a critical role in lipid metabolism by transporting excess cellular cholesterol from the peripheral tissues to the liver^1^. ApoA-I contains 11/22-mer tandem repeats that have a high tendency to form amphipathic α-helices, which have lipid-binding activity^2^. About 50% of the apoA-I secondary structure consists of α-helices, and its N-terminus comprises an α-helix bundle^3^,^4^. Some apoA-I mutants are associated with the hereditary amyloidosis called AApoA1 amyloidosis^5^. As of today, 19 mutations have been associated with AApoA1 amyloidosis^6^. One of these mutations, a G26R single substitution (herein referred to as the Iowa mutation), is the first mutation that was determined to be associated with AApoA1 amyloidosis^7^,^8^. This mutation facilitates proteolysis of apoA-I, and the resulting N-terminal fragments (amino acid residues 1–83) of the variant apoA-I deposit as amyloid fibrils in various organs, such as the kidney, liver, and heart^6^,^7^,^9^. In protein misfolding diseases such as amyloidosis, protein aggregates that accumulate or deposit in organs and tissues are abnormally folded; these aggregates are formed by aberrant production of precursor proteins or disturbances in the intracellular or extracellular protein degradation pathways^10^,^11^. The mechanism by which these apoA-I variants can form amyloid fibrils is not fully understood. Small intermediates or soluble oligomers that are found during the aggregation process are synaptotoxic or cytotoxic in some protein misfolding diseases such as Alzheimer’s disease, Parkinson’s disease, and Huntington’s disease^12^, and amyloid fibrils are also reportedly cytotoxic^13^. We previously showed that cellular interactions with and cytotoxicity of apoA-I fibrils depended on the sulfate moieties of heparan sulfate on the cell surface^14^, but the mechanisms of cellular degradation and cytotoxicity of apoA-I fibrils remain to be elucidated.

Since discovery of lysosomes in the early 1950 s, they have been shown to be involved in various lysosomal storage diseases and other human diseases such as cancer, obesity, and neurodegeneration^15^,^16^. With regard to amyloidosis, Liu *et al*. reported that amyloid β (Aβ) that has accumulated in lysosomes destabilizes the lysosomal membrane and induces neurotoxicity^17^. Another study suggested that β_2_-microglobulin amyloid fibrils accumulated in lysosomes in a neuroblastoma cell line and disrupted lysosomal membrane protein trafficking and lysosomal degradation of proteins^18^.

Macroautophagy (referred to here as autophagy) is a cellular protein degradation system for long-lived proteins and organelles^19^. Autophagy includes a number of steps: sequestration, transport or fusion to lysosomes, degradation, and reuse of degradation products. During degradation, autophagosome components that formed during the first step of autophagy are degraded by lysosomal hydrolases^20^. Thus, clearance of autophagosomes depends on lysosomal function^21^. Autophagy has also been implicated in clearance of various amyloidogenic proteins. For example, recent studies showed that autophagy played a critical role in degradation of human islet amyloid polypeptide (IAPP) and protected β-cells against cytotoxicity of IAPP^22^,^23^,^24^. Lysosome-dependent dysregulation of autophagy was also suggested to underlie cardiomyopathy pathogenesis in amyloidogenic light chain amyloidosis^25^.

Here, we used cell-based assays to investigate the role of lysosomes and autophagy in cellular degradation and cytotoxicity of G26R apoA-I fibrils (apoA-I_Iowa_ fibrils). ApoA-I_Iowa_ fibrils were degraded via the autophagy-lysosomal pathway in human embryonic kidney (HEK) 293 cells, which resulted in a loss of lysosomal acidity and in the cytosolic release of the lysosomal protease cathepsin B. The mitochondrial dysfunction caused by apoA-I_Iowa_ fibrils was reversed by enhancing the degradation of apoA-I_Iowa_ fibrils and inhibiting cathepsin B. Our results thus emphasize the importance of autophagy and lysosomes in the pathology of AApoA1 amyloidosis.

## Results

### ApoA-I_Iowa_ fibrils were degraded in an autophagy- and lysosome-dependent manner

**Figure 1:**
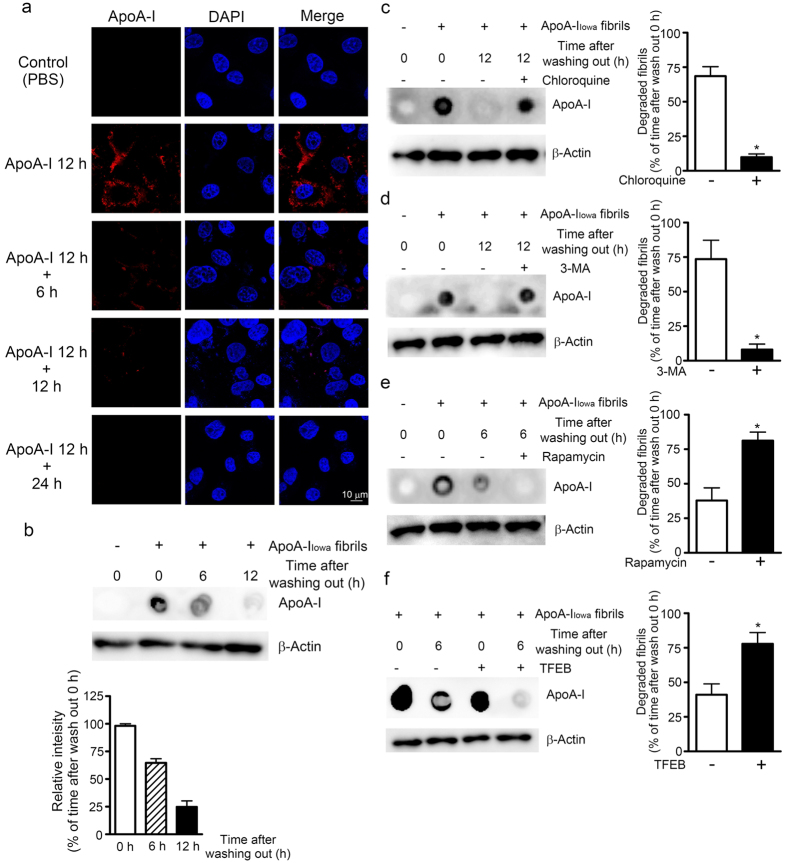
ApoA-I_Iowa_ fibrils were degraded via the autophagy-lysosomal pathway. (**a**) HEK293 cells were plated on poly-L-lysine-coated cover glasses and treated with apoA-I_Iowa_ fibrils (1 μM) at 37 °C for 12 h. Cells were washed with fresh DMEM and then incubated in DMEM at 37 °C for 0, 6, 12, or 24 h. ApoA-I_Iowa_ fibrils were visualized by using an anti-apoA-I antibody and an Alexa Fluor 568-conjugated secondary antibody. (**b**) HEK293 cells were treated with apoA-I_Iowa_ fibrils (1 μM) at 37 °C for 12 h. Cells were washed with fresh DMEM and then incubated in DMEM for 6 h, after which whole cell lysates were prepared. The apoA-I contents of the whole cell lysates were analyzed by means of dot blotting. β-Actin was used as a loading control. The graph shows quantification of cellular apoA-I_Iowa_ fibrils. Data are means ± SE of three independent experiments. (**c**,**d**,**e**) HEK293 cells were plated and treated with 1 μM apoA-I_Iowa_ fibrils for 12 h. Cells were washed with fresh DMEM and cultured at 37 °C for an additional 6 or 12 h in fresh DMEM in the presence or absence of chloroquine (**c**; 50 μg/mL, 12 h), 3-MA (**d**; 0.75 μg/mL, 12 h), or rapamycin (e; 2.5 μM, 6 h), after which whole cell lysates were prepared. The apoA-I contents of the whole cell lysates were analyzed by using dot blotting. β-Actin was used as a loading control. (**f**) Effects of TFEB overexpression on cellular degradation of apoA-I_Iowa_ fibrils. HEK293 cells were plated, transfected with pEGFP-N1-TFEB or an empty vector, and cultured at 37 °C for 48 h. Cells were treated with 1 μM apoA-I_Iowa_ fibrils for 12 h, washed with fresh DMEM, and incubated for 12 h, after which whole cell lysates were prepared. The apoA-I contents in whole cell lysates were analyzed by dot blotting with an anti-apoA-I antibody. β-Actin was used as a loading control. The graphs show quantification of the degraded fibrils. Data are means ± SE of three independent experiments. *p = 0.0013 (**c**), 0.011 (**d**), 0.017 (**e**), and 0.032 (**f**) versus each drug or TFEB (−) cells.

We first determined whether apoA-I_Iowa_ fibrils were degraded in cultured cells. As previously reported, the 1–83 fragments of wild-type (WT) apoA-I formed fibrils and these fibrils were cytotoxic^14^,^26^. However, amyloid fibrils of WT apoA-I are reportedly associated with atherosclerotic plaques^27^, not with hereditary AApoA1 amyloidosis. In the present study, we focused on the pathological effect of amyloid fibrils of the Iowa mutant of apoA-I. As shown in Supplementary Fig. S1a, the fibril content of the apoA-I_Iowa_ fibrils preparation that were used in the present study was more than 90%. One of the authors also reported that apoA-I_Iowa_ fibrils were positive for thioflavin T and consisted of long and straight fibrils having heights of 5 to 10 nm as revealed by the atomic force microscopy^26^. ApoA-I_Iowa_ fragments without incubation at 37 °C (i. e., freshly solubilized apoA-I_Iowa_ fragments) showed no cytotoxicity in the 3-(4,5-dimethylthiazol-2-yl)-2,5-diphenyltetrazolium bromide (MTT) assay (Supplementary Fig. S1b), which enabled us to exclude a possibility of cytotoxicity of non-fibrillar apoA-I_Iowa_ fragments. HEK293 cells were incubated with apoA-I_Iowa_ fibrils for 12 h, washed 3 times with Dulbecco’s modified Eagle’s medium (DMEM), and cultured for additional tme periods (0, 6, 12, 24 h). Cells were fixed, permeabilized, and stained with an anti-apoA-I antibody. As Fig. 1a shows, cells incubated with apoA-I_Iowa_ fibrils for 12 h demonstrated robust apoA-I staining, which indicates that apoA-I_Iowa_ fibrils interacted with HEK293 cells. Signals from apoA-I_Iowa_ fibrils disappeared 6–24 h after cells were washed with PBS, in a time-dependent manner. These results suggest that HEK293 cells degraded the apoA-I_Iowa_ fibrils. Results of dot blotting confirmed that apoA-I fibrils were almost completely degraded during the additional culture period (Fig. 1b). Because previous reports showed that Aβ was degraded by lysosomes^28^,^29^, we analyzed the effect of chloroquine on cellular degradation of apoA-I_Iowa_ fibrils. Chloroquine is a weak base and reportedly impaired functions of acidic organelles, including lysosomes, by means of neutralization^30^. As seen in Fig. 1c, cellular degradation of apoA-I_Iowa_ fibrils was reduced to approximately 10% in the presence of chloroquine. Thus, it was indicated that apoA-I_Iowa_ fibrils were degraded via the lysosomal pathway.

Given the finding that protein aggregates or inclusion bodies within cells were cleared by a certain type of autophagy termed aggrephagy^31^, we then studied whether apoA-I_Iowa_ fibrils were degraded via the autophagy pathway. 3-Methyladenine (3-MA) inhibits the activity of class III phosphatidylinositol 3-kinase, which plays an essential role in the biogenesis of autophagosomes, and thereby interrupts autophagy^32^. When autophagy was inhibited by adding 3-MA, degradation of apoA-I_Iowa_ fibrils was almost completely suppressed (Fig. 1d). Rapamycin (sirolimus) is a macrocyclic antibiotic made by the bacterium *Streptomyces hygroscopicus*, which occurs in the soil of Easter Island. Rapamycin inhibits the mechanistic target of rapamycin (serine/threonine kinase) complex 1 and thus induces autophagy^33^. In contrast to the effect of 3-MA, rapamycin markedly facilitated the degradation of apoA-I_Iowa_ fibrils (Fig. 1e). These findings indicate that degradation of apoA-I_Iowa_ fibrils occurred in an autophagy-lysosomal pathway-dependent fashion in cultured cells.

### Transcription factor EB (TFEB) enhanced degradation of apoA-I_Iowa_ fibrils

Sardiello *et al*. reported that TFEB induced lysosomal biogenesis and increased degradation of pathogenic proteins such as the polyglutamine-expanded huntingtin protein, which causes Huntington’s disease^34^. Because apoA-I_Iowa_ fibrils appeared to be degraded in lysosomes, we questioned whether increasing lysosomes by overexpressing TFEB would enhance degradation of apoA-I_Iowa_ fibrils. We transfected HEK293 cells with pEGFP-N1-TFEB before treating cells with apoA-I_Iowa_ fibrils. By means of immunoblotting with an anti-lysosomal-associated membrane protein 2 (LAMP2) (Supplementary Fig. S2), which is a lysosome marker^35^, we confirmed that TFEB overexpression increased the number of lysosomes. As Fig. 1f illustrates, degradation of apoA-I_Iowa_ fibrils was greatly (approximately two times) enhanced in TFEB-transfected cells. These results suggest that TFEB overexpression facilitated apoA-I_Iowa_ fibril degradation by increasing the number of lysosomes.

### Effects of dynamin and actin dynamics on cell interactions with apoA-I_Iowa_ fibrils

**Figure 2:**
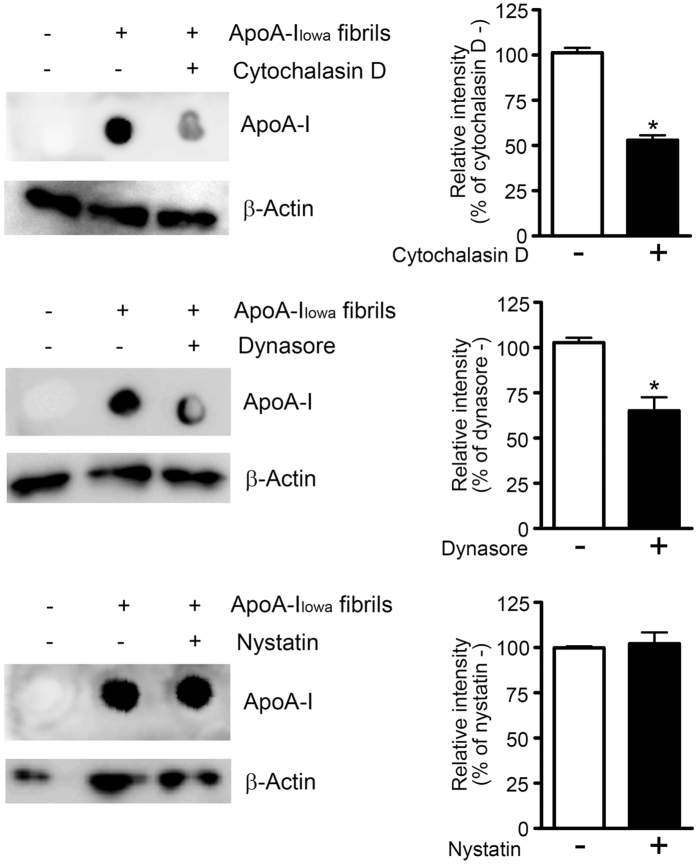
Effects of endocytic inhibition on cellular uptake of apoA-I_Iowa_ fibrils. HEK293 cells were plated and treated with 1 μM apoA-I_Iowa_ fibrils for 12 h in the presence or absence of cytochalasin D (1 μM), dynasore (15 μM), or nystatin (50 μg/mL), after which whole cell lysates were prepared. The apoA-I contents in the lysates were analyzed by using dot blotting. β-Actin was used as a loading control. The graphs show quantification of cellular apoA-I_Iowa_ fibrils. Data are means ± SE of three independent experiments. *p = 0.0002 (cytochalasin D) and 0.0098 (dynasore) versus each drug (−) cells.

The above-described results showing that apoA-I_Iowa_ fibrils were degraded via the autophagy-lysosomal pathway strongly suggested that apoA-I_Iowa_ fibrils were internalized by cells and transported to lysosomes. Thus, we studied the internalization pathway of apoA-I_Iowa_ fibrils by using HEK293 cells and inhibitors of different types of endocytosis. Inasmuch as many endocytosis pathways depend on actin dynamics^36^, we also used cytochalasin D, which inhibits actin dynamics-dependent endocytosis by disrupting actin polymerization^37^. Dynasore is a small molecule that inhibits dynamin 2, which is essential for endocytic vesicle formation in clathrin- and caveolin-mediated endocytosis, as well as clathrin- and caveolin-independent endosytosis^38^. Nystatin disrupts caveolae formation by binding to sterols and thus inhibits caveolin-mediated endocytosis. Cytochalasin D and dynasore, but not nystatin, reduced the content of apoA-I_Iowa_ fibrils in HEK293 cells by 40–50% after incubation (Fig. 2). These results suggest that dynamin and actin dynamics were involved in internalization of apoA-I_Iowa_ fibrils.

### ApoA-I_Iowa_ fibrils induced lysosomal and mitochondrial dysfunction

**Figure 3:**
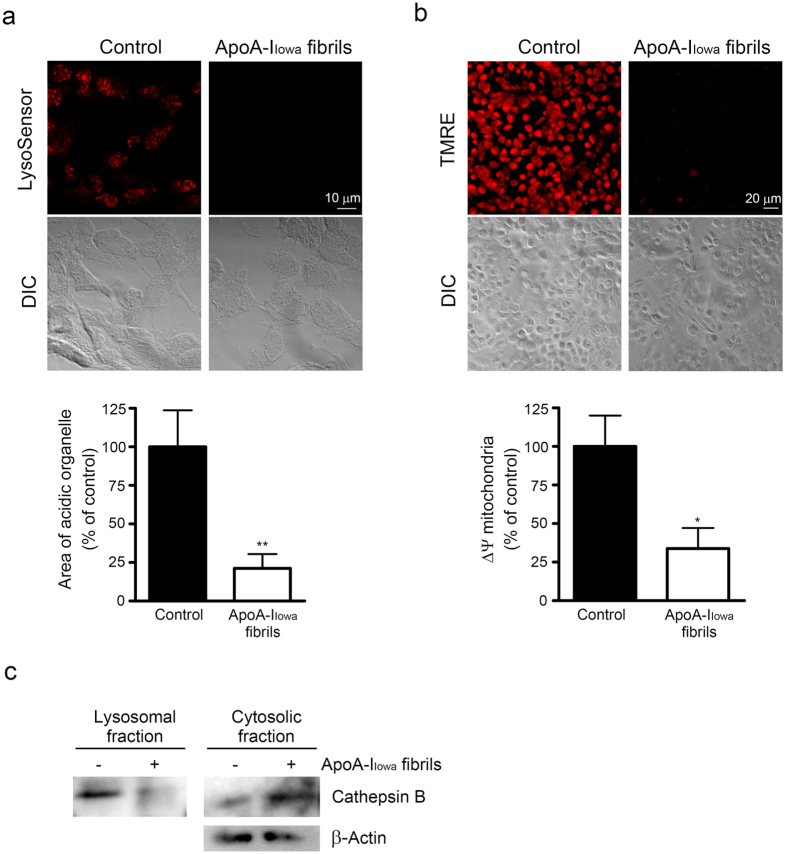
ApoA-I_Iowa_ fibrils induced lysosomal dysfunction, mitochondrial depolarization, and cytosolic release of cathepsin B. (**a**) HEK293 cells were plated on poly-L-lysine-coated cover glasses and treated with 1 μM apoA-I_Iowa_ fibrils at 37 °C for 6 h. The number of acidic organelles was assessed by using LysoSensor dye. Representative images of acidic signals (red) and differential interference contrast (DIC) microscopy are shown. The graph shows quantified LysoSensor signals. Data are means ± SE of three independent experiments. **p = 0.0093 versus non-treated cells. (**b**) Mitochondrial membrane potential was analyzed by using TMRE fluorescent dye, after treatment with 1 μM apoA-I_Iowa_ fibrils for 12 h. Representative images of TMRE fluorescence (red) and DIC microscopy are shown. The graph shows quantified TMRE signals. Data are means ± SE of three independent experiments. *p = 0.016 versus non-treated cells. (**c**) The lysosomal and cytosolic fractions of HEK293 cells that had been treated with 1 μM apoA-I_Iowa_ fibrils for 6 h were subjected to Western blotting with an anti-cathepsin B antibody to assess cytosolic release of cathepsin B. β-Actin was used as a loading control. The data represent two independent experiments.

Because some amyloid fibrils reportedly interfered with lysosomal function^18^,^25^ and our data strongly suggested that apoA-I_Iowa_ fibrils were transported to lysosomes, we investigated whether apoA-I_Iowa_ fibrils would affect lysosomal function. We used LysoSensor, a pH-sensitive fluorescent dye, to assess lysosomal pH. Consistent with our previous study with Chinese hamster ovary cells^14^, the numbers of acidic compartments including lysosomes decreased in HEK293 cells treated with apoA-I_Iowa_ fibrils (Fig. 3a), which suggests that apoA-I_Iowa_ fibrils caused a reduction in lysosomal acidity.

We also previously showed that apoA-I_Iowa_ fibrils caused increased production of reactive oxygen species (ROS)^39^. Lysosomal membrane permeabilization and subsequent cytosolic release of lysosomal protease may lead to a loss of mitochondrial membrane potential and ROS production^40^. We found, as Fig. 3b illustrates, that apoA-I_Iowa_ fibrils induced a loss of mitochondrial polarization in HEK293 cells. These findings, together with results showing that apoA-I_Iowa_ fibrils were transported to lysosomes and caused lysosomal dysfunction, indicated the possibility that apoA-I_Iowa_ fibrils caused lysosomal membrane permeabilization.

Cathepsin B is a cysteine cathepsin^41^ that stimulates ROS production and has a role in the loss of mitochondrial inner membrane potential after its release into cytosol^42^. We thus evaluated whether apoA-I_Iowa_ fibrils would induce cytosolic release of cathepsin B. Figure 3c shows that cathepsin B in the cytosolic fraction increased in cells that were treated with apoA-I_Iowa_ fibrils, which suggests that apoA-I_Iowa_ fibrils induced leakage of lysosomal cathepsin B into the cytosol. As shown in Supplementary Fig. S3, we did not observed TUNEL (terminal deoxynucleotidyl transferase dUTP nick end labeling) positive cells in apoA-I_Iowa_ fibril-treated cells.

### Rescue of HEK293 cells from cytotoxicity of apoA-I_Iowa_ fibrils

**Figure 4:**
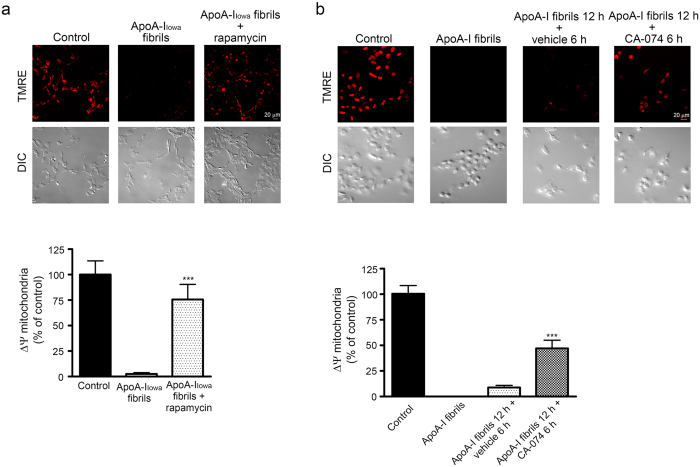
Rapamycin and an inhibitor of cathepsin B improved mitochondrial depolarization induced by apoA-I_Iowa_ fibrils. HEK293 cells were plated on poly-L-lysine-coated cover glasses and treated with 1 μM apoA-I_Iowa_ fibrils at 37 °C for 12 h (**a**) in the presence or absence of rapamycin (2.5 μM) or (**b**) in the presence or absence (vehicle) of an inhibitor of cathepsin B (CA-074, 1 μM) for 6 h for recovery from the cytotoxicity of apoA-I_Iowa_ fibrils, after which mitochondrial membrane potential was analyzed by using TMRE dye. Representative images of TMRE fluorescence (red) and DIC microscopy are shown. The graphs show quantification of TMRE signals. Data are means ± SE of three independent experiments. Where no bars appear in the graphs, experimental values were between 0 and 0.013. (**a**) ***p = 0.00032 versus cells treated with apoA-I_Iowa_ fibrils. (**b**) ***p = 0.000070 versus apoA-I_Iowa_ fibrils for 12 h or vehicle for 6 h.

We also investigated whether enhancement of degradation of apoA-I_Iowa_ fibrils would rescue cells that were damaged by the fibrils. Because rapamycin facilitated degradation of apoA-I_Iowa_ fibrils, we incubated cells in the presence of rapamycin and assessed mitochondrial dysfunction. Rapamycin lessened the mitochondrial dysfunction caused by treating cells with apoA-I_Iowa_ fibrils (Fig. 4a). Next, to investigate that release of cathepsin B into the cytosol was responsible for mitochondrial depolarization, we treated cells with CA-074, which is a cathepsin B inhibitor^43^, after treating them with apoA-I_Iowa_ fibrils. The additional treatment with CA-074 ameliorated the apoA-I_Iowa_ fibril-induced mitochondrial dysfunction (Fig. 4b), which suggests that activity of cytosolic cathepsin B contributed to apoA-I_Iowa_ fibril-induced mitochondrial dysfunction.

### ApoA-I_Iowa_ fibrils impaired autophagic clearance

**Figure 5:**
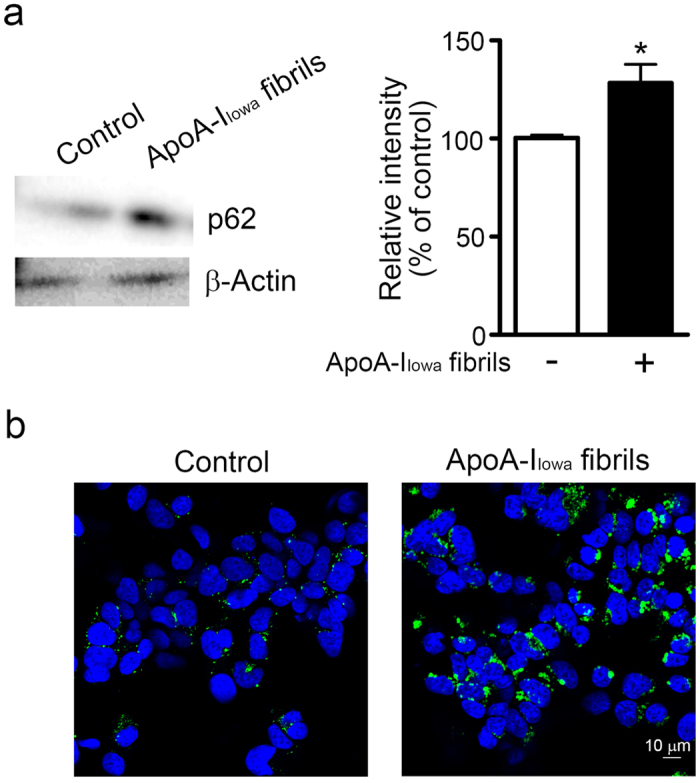
ApoA-I_Iowa_ fibrils inhibited autophagic flux in HEK293 cells. (**a**) HEK293 cells were plated and treated with 1 μM apoA-I_Iowa_ fibrils at 37 °C for 12 h, after which whole cell lysates were prepared. The lysates were subjected to Western blotting with an anti-p62 antibody. β-Actin was used as a loading control. The graph shows quantification of p62. Data are means ± SE of three independent experiments. *p = 0.042 versus non-treated cells. (**b**) Cells were plated on poly-L-lysine-coated cover glasses and treated with 1 μM apoA-I_Iowa_ fibrils at 37 °C for 12 h. After cells were fixed, they were stained with an anti-p62 antibody, followed by an Alexa Fluor 488-conjugated secondary antibody. DAPI counterstaining appears blue.

**Figure 6:**
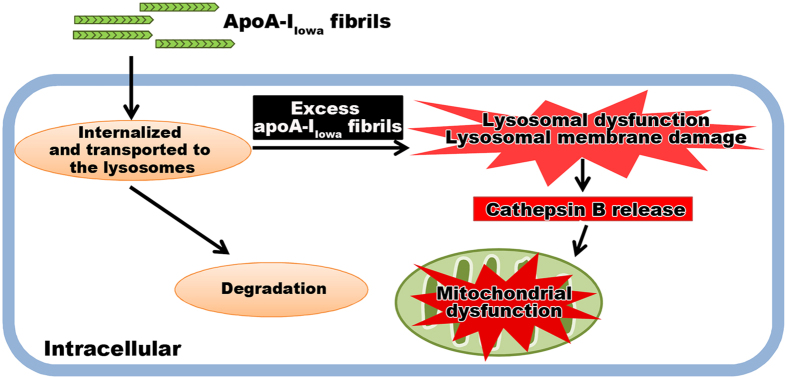
A possible mechanism of the cytotoxicity of apoA-I_Iowa_ fibrils. ApoA-I_Iowa_ fibrils were internalized and transported to the lysosomes, and some portions of them were degradable in the lysosomes. However, the presence of an excess amount of apoA-I_Iowa_ fibrils in the lysosomes caused a loss of lysosomal acidity and cytosolic release of cathepsin B followed by mitochondrial depolarization.

The p62 protein sequestosome 1 is a ubiquitin-binding scaffold protein and also binds to LC3, microtubule-associated protein 1 light chain 3, which is a specific autophagy effector^44^. p62 is an autophagy adaptor molecule and is used as a marker of autophagic flux, inasmuch as p62 is degraded in the autolysosome, which is a product of direct fusion of an autophagosome and a lysosome^45^. Because apoA-I_Iowa_ fibrils caused a loss of lysosomal acidity, we evaluated whether lysosomal activity also decreased, which would lead to accumulation of p62. As Fig. 5a shows, apoA-I_Iowa_ fibrils induced an increase in p62 protein level. Immunocytochemistry demonstrated swollen p62-positive puncta in apoA-I_Iowa_ fibril-treated cells (Fig. 5b). These data, together with findings that apoA-I_Iowa_ fibrils induced lysosomal dysfunction, suggest that apoA-I_Iowa_ fibrils impaired clearance of p62 protein by interfering with lysosomal proteolytic activity. A summary of our findings is shown in Fig. 6.

## Discussion

The lysosomal pathway has been shown to have an important role in degradation of protein aggregates including those of α-synuclein, tau, and mutant huntingtin proteins^46^ as well as IAPP^22^,^23^,^24^. Autophagy has been implicated in the clearance of protein aggregates in neurodegenerative diseases^47^,^48^. In the present study, degradation of apoA-I_Iowa_ fibrils occurred via the autophagy-lysosomal pathway. Upregulation of lysosomal biogenesis by TFEB overexpression facilitated this degradation. Furthermore, apoA-I_Iowa_ fibrils induced lysosomal membrane permeabilization and a loss of lysosomal acidity. Thus, although apoA-I_Iowa_ fibrils were degraded in lysosomes, the presence of an excess number of apoA-I_Iowa_ fibrils in the lysosomes, more than the lysosomes could degrade, may be detrimental to the lysosomes. As p62 was accumulated in the apoA-I_Iowa_ fibrils-treated cells, it is also suggested that the proteolytic capacity of the lysosomes was disturbed. One study showed that inhibition of mammalian target of rapamycin, mTOR, resulted in induction of defective autophagy and exacerbated neurodegenerative phenotypes in amyotrophic lateral sclerosis^49^. In contrast, Guan *et al*. showed that targeting mTOR by rapamycin protected cardiomyocytes against amyloidogenic light chain proteotoxicity^25^. In agreement with this finding, our study here demonstrated that rapamycin reduced mitochondrial dysfunction induced by apoA-I_Iowa_ fibrils. All these data suggest that the autophagy-lysosomal pathway plays a critical role in the clearance of apoA-I fibrils and that targeting of autophagy and lysosomes may be an attractive strategy for treatment of AApoA1 amyloidosis. Jakhria *et al*. reported that fragmented amyloid fibrils of β_2_-microglobulin accumulated in lysosomes and impaired their proteolytic ability without causing an increase in lysosomal pH^18^, whereas amyloidogenic light chain proteins impaired autophagic flux by increasing lysosomal pH and subsequently interfering with the proteolytic ability of the lysosomes^25^. In the present study, an overload of lysosomes with apoA-I_Iowa_ fibrils as well as an increase in lysosomal pH may contribute to impaired lysosomal proteolytic activity.

We previously showed that apoA-I_Iowa_ fibrils inhibited the reduction of 3-(4,4-dimethylthiazol-2-yl)-2,5-diphenyltetrazolium bromide (MTT) and induced ROS production^14^,^39^. Also, apoA-I_Iowa_ fibrils induced mitochondrial depolarization^14^. These results suggested that apoA-I_Iowa_ fibrils acted, directly or indirectly, on mitochondria. Our results showing that apoA-I_Iowa_ fibrils were transported to lysosomes and induced increased lysosomal acidity also suggest that apoA-I_Iowa_ fibrils may act on lysosomes from the inside. Thus, apoA-I_Iowa_ fibrils induced cytosolic release of the lysosomal protease cathepsin B, which is reportedly responsible for mitochondrial depolarization^42^. Indeed, mitochondrial depolarization induced by apoA-I_Iowa_ fibrils was rescued by treating cells with a cathepsin B inhibitor. A previous study has shown that Aβ42 induced lysosomal membrane damage and cytosolic release of lysosomal contents in cultured cell^17^. Our results indicate that lysosomal dysfunction and cytosolic release of lysosomal contents are also crucial for cytotoxicity of apoA-I_Iowa_ fibrils. How apoA-I_Iowa_ fibrils induce lysosomal membrane damage is currently unknown. Milanesi *et al*. reported that amyloid fibrils formed from β_2_-microglobulin interacted strongly with and disrupted lipid bilayers^50^, and disruption of membranes was enhanced in acidic pH^51^. One of the authors of the present study recently reported that formation of apoA-I_Iowa_ fibrils was promoted on a lipid membrane, which suggests that apoA-I_Iowa_ fibrils may interact with lipid bilayers^52^. Thus, apoA-I_Iowa_ fibrils may interact with and disrupt the lysosomal membrane in a manner similar to that of β_2_-microglobulin amyloid fibrils. As we did not observed cell death in apoA-I_Iowa_ fibril-treated cells, we ruled out a possibility that cytosolic release of lysosomal cathepsin B was due to cell death. Although ROS production was linked to mitochondrial depolarization^53^,^54^ and oxidative stress induced lysosomal labilization^55^, our results indicate that the primary inducer of mitochondrial dysfunction in apoA-I_Iowa_ fibril-treated cells was lysosomal membrane permeabilization. Lysosomal membrane permeabilization may also contribute to the loss of lysosomal acidity.

TFEB is a basic helix-loop-helix leucine zipper transcription factor and was identified as a master regulator of lysosomal genes including LAMP1, LAMP2, cathepsins, and subunits of vacuolar ATPases^34^. TFEB overexpression in cultured cells increased lysosomal biogenesis and enhanced clearance of pathogenic protein aggregates formed by the mutant huntingtin protein^34^. Astrocytic expression of TFEB reportedly facilitated Aβ clearance and attenuated amyloid plaque formation in a mouse model of Alzheimer’s disease^56^. In our study here, TFEB overexpression promoted degradation of apoA-I_Iowa_ fibrils. Thus, activation of lysosomal biogenesis may be a potential therapeutic target and enhance cell clearance of protein aggregates in neurodegenerative diseases as well as amyloidosis.

Pinocytosis-dependent and dynamin-dependent endocytoses were implicated in the internalization and cytotoxicity of β_2_-microglobulin amyloid fibrils^18^,^57^. In the present study, cytochalasin D and dynasore inhibited degradation of apoA-I_Iowa_ fibrils, whereas nystatin did not. Cytochalasin D and nystatin are inhibitors of endocytosis that depends on actin dynamics and caveolae formation, respectively^36^,^38^. Dynasore is an inhibitor of the GTPase activity of dynamin, which is a regulator of membrane fission and indispensable for clathrin- and caveolin-mediated endocytosis, as well as clathrin- and caveolin-independent endocytosis^37^. Thus, our results suggest that apoA-I_Iowa_ fibrils may be internalized by cells and transported to lysosomes in a dynamin- and actin dynamics-dependent, but caveolin-independent, manner. Similarly, dynasore and cytochalasin D reportedly reduced the cytotoxicity of β_2_-microglobulin amyloid fibrils, possibly by inhibiting fibril internalization^18^,^57^. Nonphagocytic cells internalized beads having a diameter of 5.5 μM in a clathrin- and dynamin-dependent fashion^58^. Given that some types of pinocytosis depend on dynamin^36^, apoA-I_Iowa_ fibrils may be internalized via pinocytosis and/or dynamin-dependent endocytosis. Elucidating the detailed mechanisms of internalization and intracellular transport of amyloid fibrils is an important challenge for the future.

In summary, we showed that apoA-I_Iowa_ fibrils were degraded via the autophagy-lysosomal pathway and induced lysosomal and mitochondrial dysfunction. Degradation of apoA-I_Iowa_ fibrils was facilitated by enhancing autophagy and lysosomal biogenesis, and cytotoxicity of apoA-I_Iowa_ fibrils was consequently ameliorated. These results indicate that enhancing the degradation of apoA-I_Iowa_ fibrils may be a therapeutic strategy. Although additional studies are needed to confirm the role of the autophagy-lysosomal pathway in clearance of apoA-I fibrils *in vivo*, our findings support the importance of the autophagy-lysosomal pathway in the pathogenesis and pathology of amyloidosis and provide a new insight into the development of AApoA1 amyloidosis treatment.

## Materials and Methods

### Materials

Chloroquine was purchased from Wako Pure Chemical Industries (Osaka, Japan). 3-MA, rapamycin, and dynasore were purchased from Cayman Chemical (Ann Arbor, MI). A polyclonal anti-p62 antibody was purchased from Cell Signaling Technology, Inc. (Beverly, MA), a polyclonal anti-β-actin antibody was from Sigma (St. Louis, MO), and a polyclonal anti-LAMP2 antibody was from Bioss Antibodies (Woburn, MA). An inhibitor of cathepsin B (CA-074) was purchased from Peptide Institute, Inc. (Osaka, Japan). Nystatin and cytochalasin D were purchased from Sigma. A monoclonal anti-apoA-I antibody (Wt20-7) was produced as previously described^14^. pEGFP-N1-TFEB was a gift from Shawn Ferguson (Addgene plasmid #38119)^59^.

### Preparation of apoA-I proteins

cDNA that encodes the N-terminal fragment (amino acid residues 1–83) of apoA-I was obtained by using PCR methods in which full-length human apoA-I cDNA was used as the template. A mutation to create the G26R variant was introduced into the cDNA encoding the N-terminus of apoA-I (amino acid residues 1–83) by using the QuikChange Site-Directed Mutagenesis Kit (Stratagene, La Jolla, CA). The cDNA was ligated into the pET32a+ expression vector (Novagen, Madison, WI), after which the construct was transformed into *Escherichia coli* strain BL21 Star (DE3) (Thermo Fisher Scientific, Waltham, MA). The apoA-I fusion proteins were expressed and purified as previously described^4^. The apoA-I preparations were at least 95% pure, as determined by means of sodium dodecyl sulfate-polyacrylamide gel electrophoresis (SDS-PAGE) followed by staining with Coomassie Brilliant Blue. The apoA-I preparations were lyophilized and stored at −20 °C before use.

### Preparation of apoA-I fibrils

The N-terminal fragment (amino acid residues 1–83) of apoA-I carrying the Iowa mutation was solubilized in 6 M guanidine hydrochloride in phosphate-buffered saline (PBS) and dialyzed into PBS. The peptide solution obtained was diluted with PBS to give a final concentration of 0.3 mg/mL, and the solution was incubated in a microcentrifugation tube in a rotating mixer at 37 °C for 7 days. Fibril formation of the apoA-I_Iowa_ fragments were monitored by measuring fluorescence of thioflavin T (10 μM) at 485 nm with an excitation wavelength of 445 nm.

### Cell culture

HEK293 cells were cultured in DMEM (Sigma) supplemented with 10% heat-inactivated fetal bovine serum (BIOWEST SAS, Nuaillé, France), 100 U/mL penicillin (Sigma), and 100 μg/mL streptomycin (Sigma) at 37 °C in an atmosphere containing 5% CO_2_.

### Assay of cellular degradation of apoA-I_Iowa_ fibrils by using dot blotting and immunocytochemistry

Cells were plated on 6-well culture plates and cultured for 12 h. Cells were then incubated with 1 μM apoA-I_Iowa_ fibrils at 37 °C for 12 h, followed by an additional incubation in fresh DMEM with or without inhibitors or inducers of lysosomes and autophagy. In some experiments, cells were transfected with the pEGFP-N1 plasmid containing human *TFEB* cDNA by using the ViaFect Transfection Reagent (Promega Corporation, Fitchburg, WI) and were cultured for 48 h before treatment with apoA-I_Iowa_ fibrils. After incubation, whole cell lysates were prepared by trichloroacetic acid precipitation and apoA-I contents were analyzed by using dot blotting as previously described^14^. Briefly, cells were washed 3 times with PBS and then treated with 10% trichloroacetic acid (w/v) in PBS. After incubation on ice for 30 min, the samples were centrifuged at 1,000 × g for 5 min at 4 °C. Resultant precipitates were dissolved in SDS-PAGE sample buffer [0.125 M Tris-HCl, 4% (w/v) SDS, 20% (v/v) glycerol, and 0.01% (w/v) bromophenol blue] for preparation of whole cell lysates. The whole cell lysates obtained were blotted on nitrocellulose membranes (Pall Corporation, Port Washington, NY). ApoA-I_Iowa_ fibrils on the membranes were probed with an anti-apoA-I antibody followed by a horseradish peroxidase-labeled anti-mouse antibody (Cell Signaling Technology, Inc.) and ImmunoStar LD (Wako Pure Chemical Industries). Protein contents of cell lysates were normalized to the expression level of β-actin protein. Signals were visualized and analyzed by using an LAS-3000 luminescent image analyzer (Fujifilm, Tokyo, Japan).

For immunocytochemistry, cells were plated on a poly-L-lysine-coated cover glass and cultured overnight. They were then incubated with 1 μM apoA-I_Iowa_ fibrils at 37 °C for 12 h, after which they were washed with fresh DMEM and cultured in DMEM for an additional 6–24 h. The cells were fixed with 4% paraformaldehyde in PBS at room temperature for 20 min. After the cells were washed 3 times with PBS, they were blocked and permeabilized with 10% normal goat serum and 0.05% saponin in PBS at room temperature for 20 min. They were then incubated with an anti-apoA-I antibody followed by Alexa Fluor 568-conjugated secondary antibody (Thermo Fisher Scientific). The stained specimens were mounted with Vectashield mounting medium containing 4′,6-diamidino-2-phenylindole (DAPI) (Vector Laboratories, Inc., Burlingame, CA) and examined with an A1R confocal laser microscope (Nikon Corporation, Tokyo, Japan).

### Assay of the internalization pathway of apoA-I_Iowa_ fibrils via dot blotting

HEK293 cells were plated on 6-well culture plates and treated with 1 μM apoA-I_Iowa_ fibrils at 37 °C for 12 h in the presence or absence of cytochalasin D (1 μM), dynasore (15 μM), or nystatin (50 μg/mL). Whole cell lysates were prepared and the apoA-I contents of the lysates were analyzed by using dot blotting as described above.

### Analysis of autophagic flux via Western blotting and immunocytochemistry

Briefly, for Western blotting with an anti-p62 antibody, cells were plated on 6-well culture plates and treated with 1 μM apoA-I_Iowa_ fibrils at 37 °C for 12 h, after which whole cell lysates were prepared and subjected to Western blotting with an anti-p62 antibody, as described above.

For immunocytochemistry, cells were plated on a poly-L-lysine-coated cover glass and cultured overnight. The cells were then incubated with 1 μM apoA-I_Iowa_ fibrils at 37 °C for 12 h, washed with PBS, and fixed with 4% paraformaldehyde in PBS at room temperature for 20 min. After the cells were washed 3 times with PBS, they were blocked and permeabilized with 10% normal goat serum and 0.05% saponin in PBS at room temperature for 20 min. The cells were then incubated with an anti-p62 antibody followed by Alexa Fluor 488-conjugated secondary antibody (Thermo Fisher Scientific). Stained specimens were mounted with Vectashield mounting medium containing DAPI and examined with an A1R confocal laser microscope.

### Measurement of mitochondrial membrane potential

Mitochondrial membrane potential was determined by using the mitochondrial membrane potential-sensitive fluorophore tetramethylrhodamine ethyl ester (TMRE) (MitoPT assay kit; ImmunoChemistry Technologies, Bloomington, MN). Briefly, HEK293 cells were plated and cultured on a poly-L-lysine-coated cover glass, followed by treatment with 1 μM apoA-I fibrils at 37 °C for 12 h. After the cells were washed 3 times with PBS, they were treated with TMRE (10 nM) at 37 °C for 20 min in the dark. TMRE fluorescence was acquired by using an excitation wavelength of 555 nm with an LSM 710 confocal microscope (Carl Zeiss MicroImaging GmbH, Jena, Germany). For quantification, images were analyzed by using ImageJ software (NIH, Bethesda, MD).

### Assessment of lysosomal pH

Lysosomal pH was analyzed by means of LysoSensor Yellow/Blue DND-160 (Thermo Fisher Scientific) as previously reported^14^. Briefly, HEK293 cells were treated with 1 μM apoA-I fibrils at 37 °C for 6 h followed by the LysoSensor (1 μM, 3 min). Samples were examined with an LSM 710 confocal laser microscope according to the manufacturer’s instructions. Red signals indicated acidic conditions. For quantification, images were analyzed by using ImageJ software.

### Assessment of cytosolic release of cathepsin B

For subcellular fractionation into cytosolic and lysosomal fractions, cells were treated with 1 μM apoA-I fibrils at 37 °C for 6 h, washed 3 times with PBS and incubated with twice the volume of MSH buffer (210 mM mannitol, 70 mM sucrose, 20 mM HEPES, 1 mM EDTA, and a protease inhibitor cocktail, pH 7.5) at 4 °C for 45 min. Cells were lysed by using a 25-G needle and were then centrifuged for 5 min at 350 × g to precipitate nuclei. To obtain a crude membrane/lysosomal fraction, the postnuclear supernatant was centrifuged at 16,000 × g for 20 min followed by ultracentrifugation at 100,000 × g for 45 min, and the pellet was resuspended in MSH buffer containing 1% Triton. The supernatant (cytosolic fraction) and lysosomal fraction were subjected to Western blotting with an anti-cathepsin B antibody as described above.

### Statistical analysis

Data were analyzed via one-way analysis of variance, including the appropriate variables, followed by the Bonferroni test or unpaired Student’s *t*-test. Results were regarded as significant for p < 0.05.

## Additional Information

**How to cite this article**: Kameyama, H. *et al*. Iowa Mutant Apolipoprotein A-I (ApoA-I_Iowa_) Fibrils Target Lysosomes. *Sci. Rep.* **6**, 30391; doi: 10.1038/srep30391 (2016).

## Supplementary Material

Supplementary Information
