# Supplementary material for: Iowa Mutant Apolipoprotein A-I (ApoA-IIowa) Fibrils Target Lysosomes
Source: Sci Rep. 2016 Jul 28;6:30391. doi: 10.1038/srep30391 (PMC4964564; doi:10.1038/srep30391)
Supplement: Supplementary Information [file srep30391-s1.doc]

**Supplementary Information**

**Iowa Mutant Apolipoprotein A-I (ApoA-I_Iowa_) Fibrils Target Lysosomes**

Hirokazu Kameyama^1,§^, Hiroyuki Nakajima^1,§^, Kazuchika Nishitsuji^2^,*, Shiho Mikawa^1,3^, Kenji Uchimura^4^, Norihiro Kobayashi^5^, Keiichiro Okuhira^1^, Hiroyuki Saito^3^, and Naomi Sakashita^2^

^1^Department of Molecular Physical Pharmaceutics, Institute of Biomedical Sciences, Tokushima University Graduate School, 1-78-1 Shomachi, Tokushima 770-8505, Japan. ^2^Department of Molecular Pathology, Institute of Biomedical Sciences, Tokushima University Graduate School, 3-18-15 Kuramoto-cho, Tokushima 770-8503, Japan. ^3^Department of Biophysical Chemistry, Kyoto Pharmaceutical University, 5 Nakauchi-cho, Misasagi, Yamashina-ku, Kyoto 607-8414, Japan. ^4^Department of Biochemistry, Nagoya University Graduate School of Medicine, 65 Tsurumai-cho, Showa-ku, Nagoya 466-8550, Japan. ^5^Department of Bioanalytical Chemistry, Kobe Pharmaceutical University, 4-19-1 Motoyama-Kitamachi, Higashinada-ku, Kobe 658-8558, Japan.


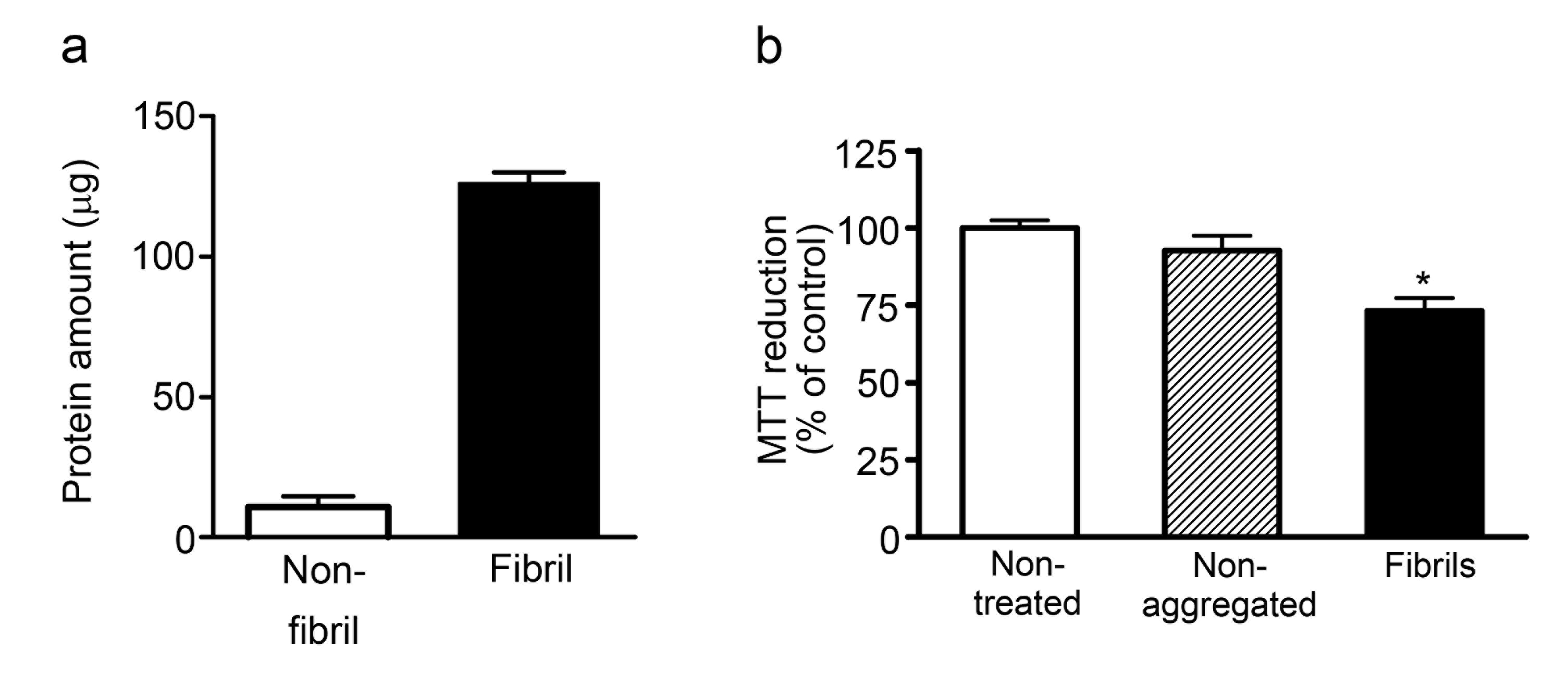


**Supplementary Figure S1.** (a) Fibril content of the apoA-I_Iowa_ fibril preparation. ApoA-I_Iowa_ fibrils were prepared as described in *Materials and methods*. Four hundred μL of the preparation was centrifuged at 20,000 × g for 40 min at 4 °C. The supernatant (non-fibril fraction) and pellet (fibril fraction) were dissolved in 4M urea, and the protein content of each fraction was measured by using the Bradford assay in which apoA-I solutions in 4M urea were used as the standard. The results are means ± SE of three independent experiments. (b) Cytotoxicity of apoA-I_Iowa_ fibrils and freshly solubilized non-aggregated apoA-I_Iowa_ fragments was assessed by means of the MTT assay as previously described^1^. The results are means ± SE of three independent experiments. *, *p* = 0.0055 versus control cells by the Dunnett test.


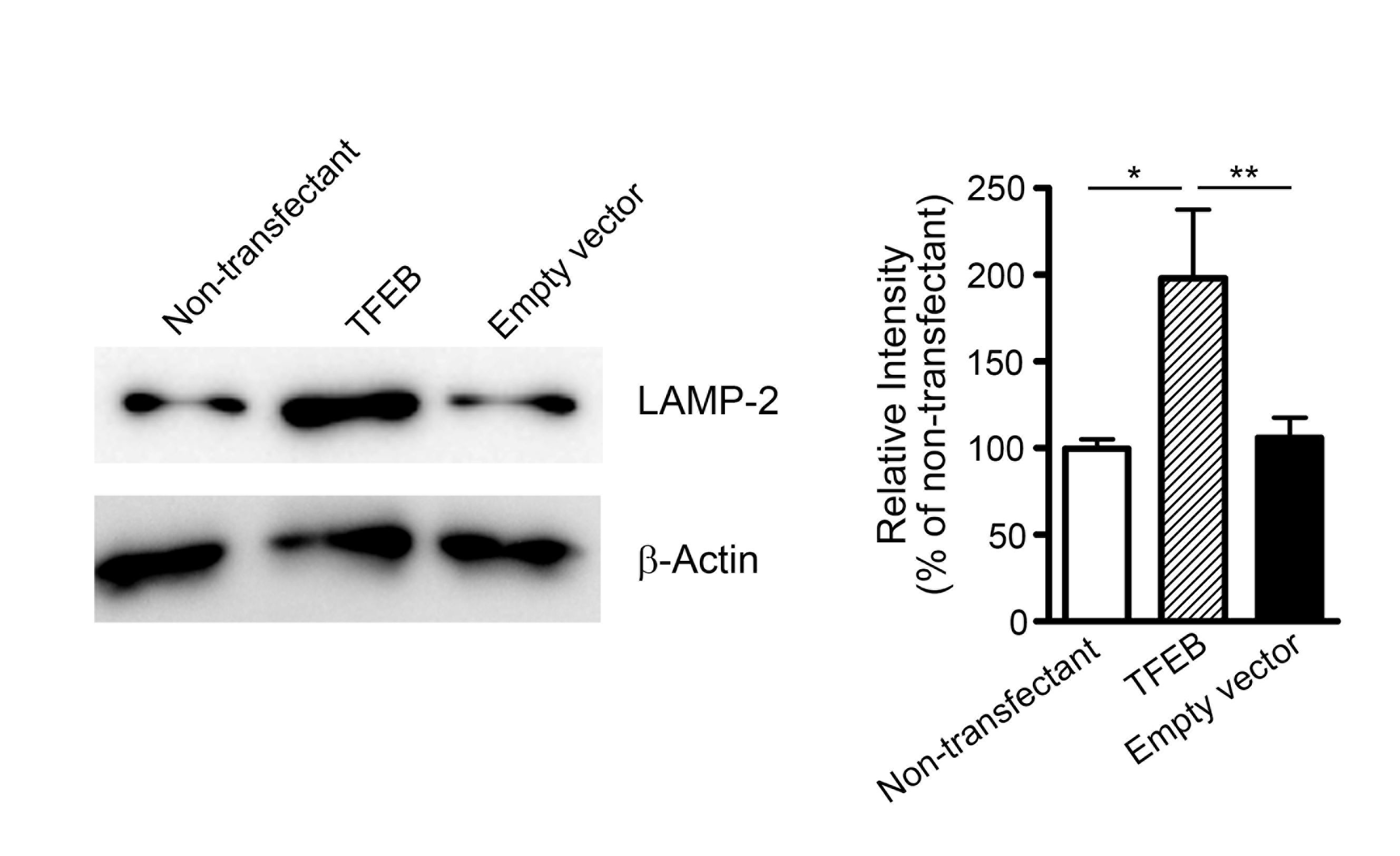


**Supplementary Figure S2.** HEK293 cells were plated, transfected with pEGFP-N1-TFEB or an empty vector, and cultured at 37 °C for 48 h. Cells were treated with 1 μM apoA-I_Iowa_ fibrils for 12 h, washed with fresh DMEM, and incubated for 12 h, after which whole cell lysates were prepared. An immunoblot with an anti-LAMP2 antibody confirmed enhancement of lysosomal biogenesis. β-Actin was used as a loading control. The graph shows quantification of LAMP2. Data are means ± SE of three independent experiments. **p* = 0.028 versus non-transfectant, ***p* = 0.035 versus empty vector by the Bonferroni test.


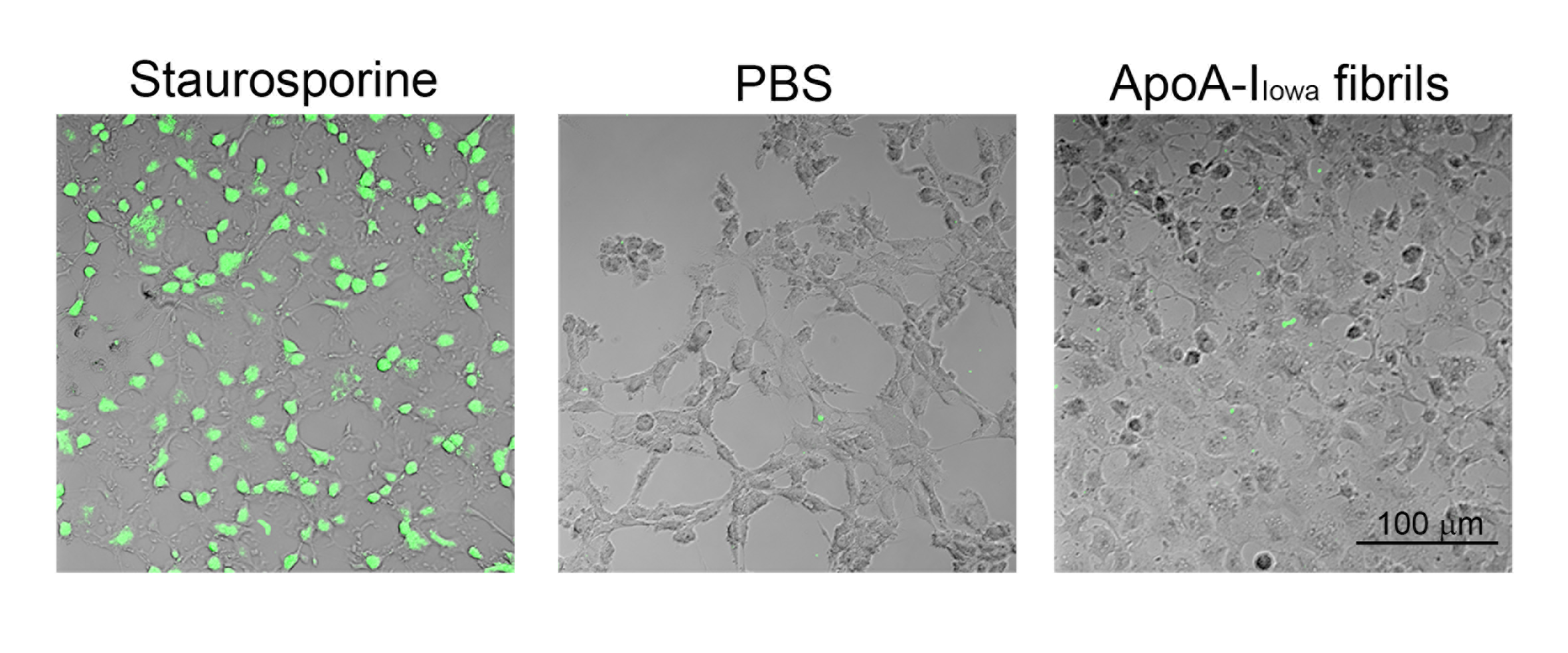


**Supplementary Figure S3.** TUNEL (terminal deoxynucleotidyl transferase dUTP nick end labeling) assay in HEK293 cells that were treated with 1µM apoA-I_Iowa_ fibrils for 6 h. After fixation with 4% PFA at room temperature for 20 min, the cells were permeabilized and incubated with the labeling buffer containing the TdT enzyme (Takara Bio Inc., Shiga, Japan) for 60 minutes at 37 °C. The specimens were examined under an LSM710 confocal microscope. Staurosporine (Cayman Chemical, 10 µM, 12 h) was used as a positive control. Merged images of the TUNEL signals (green) overlaid on differential interference contrast images are shown.

**Supplementary References**

1. Kuwabara, K. *et al.* Cellular Interaction and Cytotoxicity of the Iowa Mutation of Apolipoprotein A-I (ApoA-I_Iowa_) Amyloid Mediated by Sulfate Moieties of Heparan Sulfate. *J Biol Chem* **290**, 24210-24221 (2015).
